# Structural variability of *E. coli* thioredoxin captured in the crystal structures of single-point mutants

**DOI:** 10.1038/srep42343

**Published:** 2017-02-09

**Authors:** Martín E. Noguera, Diego S. Vazquez, Gerardo Ferrer-Sueta, William A. Agudelo, Eduardo Howard, Rodolfo M. Rasia, Bruno Manta, Alexandra Cousido-Siah, André Mitschler, Alberto Podjarny, Javier Santos

**Affiliations:** 1 Universidad de Buenos Aires, Facultad de Farmacia y Bioquímica, Instituto de Química y Fisicoquímica Biológicas, CONICET, Junín 956, C1113AAD, Buenos Aires, Argentina; 2 Laboratorio de Fisicoquímica Biológica, Instituto de Química Biológica and Center for Free Radical and Biomedical Research, Universidad de la República, Montevideo, Uruguay; 3 Department of Integrative Biology, IGBMC, CNRS, INSERM, Université de Strasbourg, Illkirch, France; 4 Instituto de Biología Molecular y Celular de Rosario (IBR), Rosario, Santa Fe, Argentina

## Abstract

Thioredoxin is a ubiquitous small protein that catalyzes redox reactions of protein thiols. Additionally, thioredoxin from *E. coli* (EcTRX) is a widely-used model for structure-function studies. In a previous paper, we characterized several single-point mutants of the C-terminal helix (CTH) that alter global stability of EcTRX. However, spectroscopic signatures and enzymatic activity for some of these mutants were found essentially unaffected. A comprehensive structural characterization at the atomic level of these near-invariant mutants can provide detailed information about structural variability of EcTRX. We address this point through the determination of the crystal structures of four point-mutants, whose mutations occurs within or near the CTH, namely L94A, E101G, N106A and L107A. These structures are mostly unaffected compared with the wild-type variant. Notably, the E101G mutant presents a large region with two alternative traces for the backbone of the same chain. It represents a significant shift in backbone positions. Enzymatic activity measurements and conformational dynamics studies monitored by NMR and molecular dynamic simulations show that E101G mutation results in a small effect in the structural features of the protein. We hypothesize that these alternative conformations represent samples of the native-state ensemble of EcTRX, specifically the magnitude and location of conformational heterogeneity.

Thioredoxins (TRX) are small globular proteins of about 100–110 residues, which are conserved in all living organisms. They participate in multiple critical redox-reactions *via* the reversible oxidation of the active site CXXC catalytic motif^1^. Two TRX forms were described in *Escherichia coli*, Trx1 and Trx2^2^. The former, called EcTRX throughout this work, is a widely used experimental model for investigating the molecular basis of protein stability, folding and function^3^,^4^,^5^,^6^,^7^,^8^,^9^. This is a monomeric protein with a characteristic α/β topology. The three-dimensional structure of the oxidized form was determined by X-ray crystallography^10^, and the structures of both oxidized and reduced states were elucidated by NMR in solution^11^. EcTRX is a highly stable protein (Δ*G*°_NU_ ~ 8 kcal mol^−1^) and its folding mechanism most likely includes at least a high-energy intermediate state, not observed under equilibrium unfolding, in which the C-terminal α-helix (CTH, Fig. 1) is mostly unfolded^12^.

The internal motions of EcTRX were investigated in detail in a previous work^13^. The backbone dynamics of EcTRX in its native state was studied by two-dimensional ^1^H−^15^N-NMR spectroscopy by means of *T*_1_, *T*_2_-relaxation and heteronuclear-NOE experiments. Reduced and oxidized forms exhibit similar dynamic characteristics on the picosecond to nanosecond timescale. EcTRX is a mostly rigid protein, but a number of regions display higher mobility in both redox states, including: (i) the N- and C- termini of the protein; (ii) residues 20–22, corresponding to the stretch linking the first α-helix and the second β-strand; and (iii) residues 73–75 plus 93–94, lying close to the redox-active site. On the other hand, in the microsecond to millisecond timescale, only the reduced form displays a region of enhanced mobility (residues 73–75), suggesting a functional role for these motions^13^.

In a previous work^14^, we studied the fragment-complementation reaction between a segment derived from the first 93 residues of EcTRX and a set of single-point mutant peptides comprising the CTH-region (residues 94–108). In that work, the capability of this set of peptides to properly regenerate the TRX fold was studied in detail. Notably, the peptide carrying the E101G mutation, which exhibits a lower secondary structure content in isolation than the wild-type peptide, showed a significant increase in Di-fluoresceinthiocarbamyl-insulin (DiFTC-insulin) reduction activity when complexed to fragment 1–93 compared with the wild-type complex. In a subsequent work^12^, we studied the effect of the same set of mutations in the structure, stability and unfolding mechanism of the full-length protein. It is worth noting that an increase in enzymatic activity was also observed for the full-length EcTRX E101G variant towards DiFTC-insulin. In that work we identified a set of full-length mutants that exhibits (singular) the same folding mechanism, almost identical spectroscopic signatures and similar enzymatic activity by comparison with wild-type EcTRX. We found that global stability of the variants is progressively altered from 8.1 (wild-type), 7.8 (N106A), 7.4 (L94A), 7.2 (E101G) to 5.8 kcal mol^−1^ (L107A). Considering these results, we expected small perturbations in structure upon mutation, offering the possibility of revealing features of the native ensemble by subtle alteration of free-energy barriers between native substates. Even though wild-type EcTRX and mutants are rather stable and presumably rigid, certain heterogeneity characteristics of the conformational space could be identified by structure analysis at high resolution. With this aim we determined and analyzed the crystal structures of the four mutants.

We determined in this work the three-dimensional structure of three full-length variants in which the point mutations occur in the CTH (E101G, N106A and L107A), and one additional variant (L94A) where a substitution is located in the loop connecting β5-strand and CTH, using X-ray diffraction. We found that the native structure is mainly unchanged for these variants. However, in the E101G mutant, an interesting case of structural variability was observed. In this mutant, an extended, non-sequentially continuous region of the protein backbone presents two alternative traces for only one of two monomers found in the asymmetric unit. We hypothesized that alternative traces and other details observed for the variants, including cavity heterogeneity, illustrate a pre-existing equilibrium between populations in the native-state ensemble in solution. In this context, crystal packing would contribute to the stabilization of these globally similar, albeit significantly different conformations.

## Results and Discussion

### Crystallization, Structure Determination and Global Structural Features

Initially, we set out the crystallization of seven EcTRX point mutants: L94A, L99A, E101G, F102A, L103A, N106A and L107A. We were unsuccessful in crystallizing L99A, F102A and L103A, the least stable variants, although many attempts were carried out using automated robotics to screen a large number of crystallization conditions. Conformational instability of these variants might be the cause of difficulty in crystallization, as the substituted residues participate in key core interactions. Well-diffracting crystals of variants L94A, E101G, N106A and L107A were obtained, and their structures were determined by molecular replacement at high resolution (PDB: 5HR2, 5HR0, 5HR3 and 5HR1, respectively).

The four EcTRX mutants were crystallized in the oxidized form, and numerous protein-metal interactions were observed as a result of the Cu(II)-salt added for crystallization, as it was previously observed for the wild-type variant^10^,^15^,^16^. Notably, the four variants crystallized in different crystal systems (Table 1), comprising monoclinic, triclinic, orthorhombic and trigonal space groups, for L94A, E101G, N106A and L107A respectively. This fact enabled us to discuss the structural features of the EcTRX fold in the different packing arrangements.

**Figure 2:**
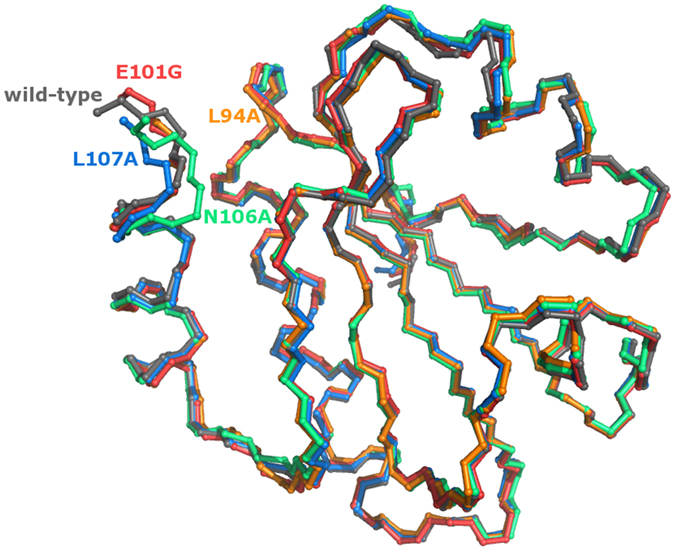
Structure superposition of Mutants and Wild-Type EcTRX. The backbone trace of the four mutants determined in this work and the wild-type are shown upon structural superposition. The name of each variant is shown in the same color as the corresponding structural model (wild-type, L94A, E101G, N106A and L107A are colored in dark gray, orange, red, green and blue, respectively). Only chain A of each variant is shown for clarity.

As expected from solution experiments^12^, the global structures of the four variants are mainly unchanged compared to wild-type EcTRX, with main-chain root-mean-square deviation of atomic position (RMSD) differences of 0.59, 0.48, 0.62 and 0.48 Å for L94A, E101G, N106A and L107A, respectively (Fig. 2). The number of protein molecules per asymmetric unit (ASU) differs among variants (Table 1). Despite being monomeric in solution, non-crystallographic symmetry is observed for the E101G and N106A mutants (two molecules per ASU) and for the L107A variant (seven molecules per ASU). Very weak electron density was observed for one of the L107A monomers, which was mostly modeled based on non-crystallographic symmetry restraints. Close examination of the resultant *F*_o_-*F*_c_ electron density maps revealed a significant negative density between cysteine residues of some disulfide bonds, and additional positive density in the opposite sides of Sγ atoms. The magnitude of this effect varies between the crystal species and the individual molecules of the corresponding ASU. Trial refinements on the most affected dataset indicated that this effect is actually an artifact of data collection, due to partial reduction by radiation damage (see Supplementary Fig. S1). Therefore, we modeled all of the cysteine pairs as disulfide bonds.

### Local Effects and Variability in the Proximity of the Mutation Sites

**Figure 3:**
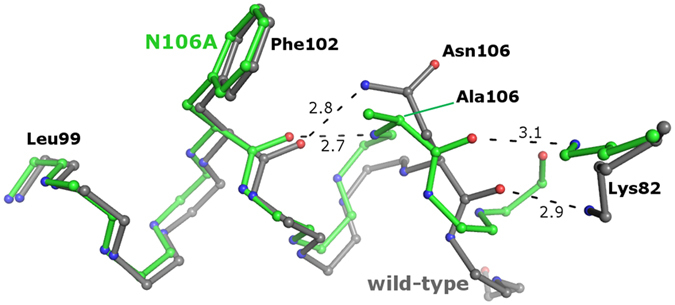
Structural Alteration upon N106A Mutation. A detailed view of the hydrogen bond between the side chain of Asn106 and the backbone of Phe102 in the wild-type variant. Instead of that, a main chain interaction between Ala106 and Phe102 is observed for the N106A mutant, with the concomitant distortion of the helix. The polar interaction of Lys82 with residue 106 is conserved in both variants.

The N106A variant was designed to eliminate a hydrogen bond linking the side-chain of Asn106 with the carbonyl oxygen of Phe102, thus N106A is expected to destabilize the native structure. In a previous work, we found that all of the native-state signatures of the N106A mutant were essentially identical to wild-type EcTRX^12^. The three-dimensional structure of this variant reveals that the hydrogen bond partner of Phe102 is now the main-chain of residue 106, through its amide nitrogen. A slight distortion of the main-chain of the CTH is produced to satisfy the hydrogen bond distance for this interaction (Fig. 3). These results are consistent with the very low effect on global stability (ΔΔ*G*°_NU_ = 0.3 kcal mol^−1^) observed for this mutation^12^. The N106A mutant exhibits a large cavity of 166 Å^2^ for chain A or 130 Å^2^ for chain B (see Supplementary Figs S2, S3 and S4). Electron density compatible with an ethanol molecule, used as a precipitant, was detected inside this cavity. More likely, the ethanol molecule establishes a hydrogen bond with Leu94. Therefore, we speculate that this cavity of EcTRX is highly dynamic, in such a way that it connects the protein core with the bulk solvent, allowing the entrance of the ethanol molecule. Considering the small difference in stability of mutant N106A compared with the wild-type, we infer that core motions (necessary to undergo the cavity rearrangements observed in N106A structure) may be shared by wild-type EcTRX. Another significant difference between wild-type and N106A is the distance between loop elements centered in positions 20 and 83, which results in the wild-type structure in a slightly “open” conformation (for instance, the distance from Cα 20 to Cα 83 is 7.4 and 4.3 Å for wild-type and N106A, respectively, Supplementary Fig. S5).

The L94A mutation was introduced to disrupt the van der Waals interactions between Leu94 and the Leu99 residue of the CTH, with a concomitant destabilization of the global structure through intramolecular packing defects. In the crystal structure of wild-type EcTRX, two small cavities of 70 and 50 Å^2^ of internal area are localized near Leu94, which coalesce in a larger cavity of 183 Å^2^ in the L94A mutant structure (see Supplementary Fig. S2). This effect approaches what can be expected from pruning the side-chain to Cβ in the wild-type structure. A decrease of the distance between loop elements centered in residues 20 and 83 is observed in this mutant in a similar manner as described for the N106A variant (Supplementary Fig. S5). No structural perturbations are found near the mutation site for the E101G and L107A mutants, which illustrate the robustness of the CTH element to sequence changes. The local effect involving loops centered in residues 20 and 83, as already mentioned, is observed for the L107A mutant and, in a lesser extension, for the E101G variant. Most likely, this “closed” conformation best represents the average native conformation of these regions and, moreover, the determined structures as a whole, allow us to capture details of the native-state ensemble. A well-preserved hydrogen bonding pattern is found in the CTH of E101G mutant, even though a Gly residue is highly destabilizing for the α-helical structure^14^. As in the case of N106, we infer that the observed cavity heterogeneity in the case of mutant E101G (Supplementary Table S1 and Fig. S2) is a feature of the EcTRX native ensemble, and an observable that is the consequence of internal fluctuations. Regarding cavities of the L107A mutant, the outcome of this substitution is a slight increase in the solvent-exposed surface, as Leu107 is a partially exposed residue.

Thereby, it was confirmed that most of the structural features of the four mutants are shared with the wild-type variant. A few local differences were found. Apart from changes involving the mutated site, these differences may represent the structural variability already present in the native state of the wild-type variant. Packing interactions may contribute to overcome energy barriers separating different substates of the native ensemble. This would enable us to study in detail the structures of samples of these underrepresented conformations, which become stabilized in the different crystal forms.

### Structural Variability of EcTRX Evidenced in the Crystal Structure of the E101G Mutant

**Figure 4:**
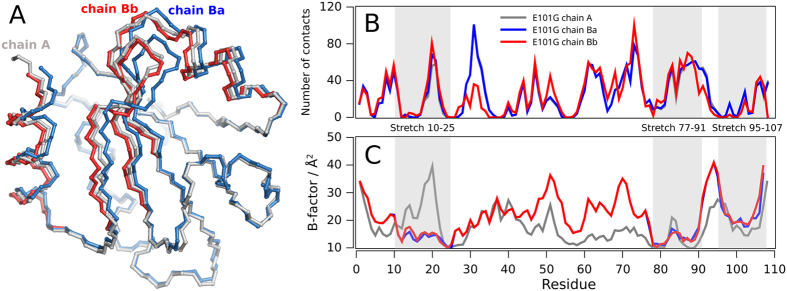
Comparison of Alternative Conformations of the E101G Variant. (**A**) The backbone of chain A is shown superimposed to chains B_a_ and B_b_. Structure alignment was performed minimizing the RMSD of backbone atoms for residues without alternative occupancies in chain B (i.e., segments 1–9 plus 26–76 and 92–94). (**B**) Crystal packing is inferred by measuring the number of inter-chain contacts per residue for each chain. (**C**) B-factors values corresponding to the Cα carbons are shown for each chain. Note that in regions without double conformations Ba superimpose with B_b_.

It was evident during the early stages of refinement of the E101G variant that a large fraction of residues of chain B (~40%), exhibits alternative traces of the backbone (Fig. 4A). No alternative traces of the protein backbone were detected for the other chain in the asymmetric unit. The affected residues belong to contiguous stretches of structure, non-sequentially continuous, compromising multiple secondary structure elements. The widespread alternative-conformation region involves residues: (i) Asp10 to Val25 (including helix 1 and a half of strand β2) (ii) Thr77 to Val91 (comprising strands β4 and β5); and (iii) Ser95 to Leu107 (involving the complete CTH). Occupancies of these conformations equals 50%, and no clashes or packing defects result when alternatively occupied segments are assigned to different single-conformation models. Then, it is difficult to split the refined model into single-conformation models from stereochemical considerations; however, a single concerted shift of the whole region provides the most reasonable explanation for the observed difference. We used this criterion to define two alternative conformations for chain B, which we named chain B_a_ and chain B_b_. No correlation is detected between the number of contacts established by a given residue with the nearby monomers of the crystal lattice, and the occurrence of alternative conformations for this residue (Fig. 4B). Indeed, the numbers of intermolecular contacts *per*-residue for chains A and B are essentially identical throughout the protein sequence, even if the latter exhibits alternative traces of the backbone. The RMSD between these alternative conformations is 0.65 Å for the backbone atoms and it occurs without significant changes in the radius of gyration or total accessible surface area (data not shown). Structural differences between these alternative traces are explained mostly through rigid-body displacements of structural elements, as a result of changes in dihedral angles for the first and last residues of each segment.

Additionally, the occurrence of alternative occupancies is not associated with particularly high or low B-factors values. The B-factors for both traces of chain B are essentially identical (Fig. 4C), with remarkably low values for the first segment of alternative occupancies (residues 10–25), as opposed to the same segment in chain A. This is suggestive of distinctive motions for this stretch of residues, but we cannot rule out that these differences may be due to subtle changes in the neighborhood of the crystal lattice. Nevertheless, the structural difference of conformations B_a_ and B_b_ occurs without significant changes in the number of intramolecular interactions (Table 2). A close inspection of conformations B_a_ and B_b_ reveals essentially identical hydrogen-bond networks. On the contrary, the structural differences are accomplished through the combination of slight increases or decreases in the distances of the hydrogen-bond partners. If the main-chain hydrogen bonds involving only the alternative conformation regions are taken into account, then around 40% of the hydrogen-bond lengths are decreased in the B_b_ conformer, whereas around 60% of these distances are increased.

**Figure 5:**
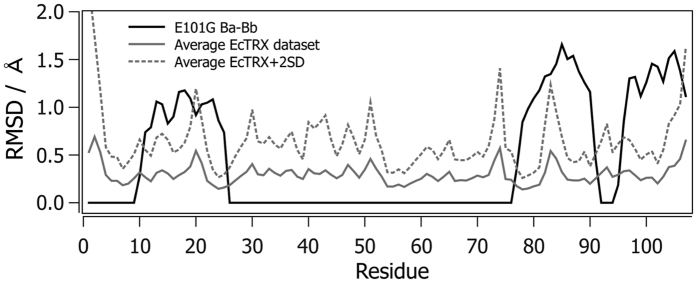
Structural Difference in Chains B of the E101G Mutant. The per-residue RMSD was calculated for the backbone atoms of chains B_a_ and B_b_ for the E101G mutant, as well as a set of 20 EcTRX available in the PDB plus the four structures determined in this work, considering only the chain A for all the structures. The average RMSD value for all of the possible pairwise alignments is shown (continuous gray line), along with the average plus two standard deviations (dashed gray line).

To investigate whether or not EcTRX regions with alternative conformations are prone to conformational variability, we conducted a search for prior evidence from published structures in the PDB. Structures of wild-type and mutant EcTRX were collected, trimmed to a single chain per variant (chain A), and highly divergent structures were removed. The resulting sets of coordinates, plus the four mutants reported in this work, were pairwise superposed and the per-residue RMSD was calculated. The results are presented in Fig. 5 along with the corresponding values for the difference between B_b_ and B_a_ traces of the E101G mutant. The segments exhibiting alternative traces are not coincident with the stretches of high structural variability in the representative set of mutants.

In assessing the structural significance of alternative traces of the EcTRX E101G variant, we wondered whether these conformations represent true low-energy conformers selected during crystallization. Preliminary information about differences in thermodynamic stability among conformations was obtained by performing *foldX*^17^ calculations for each conformation, i.e., chain A and chains B_a_ and B_b_. It should be noted that these calculations were carried out after applying the *foldX* repair protocol, an energy-minimization procedure that changes the side-chain rotamers without altering backbone conformation. Taking into account this method’s reported error of ±1 kcal mol^−1^ in free energy difference, these calculations suggest that chain B_b_ conformation might be slightly destabilized compared with chain A (Δ*G*°_NU_ = −11.94, −10.46 and −8.93 kcal mol^−1^ for chain A, B_a_ and B_b_, respectively, Supplementary Fig. S6 and Table S2), whereas the calculated difference in free energy between conformations A and B_a_ is not significant.

**Figure 6:**
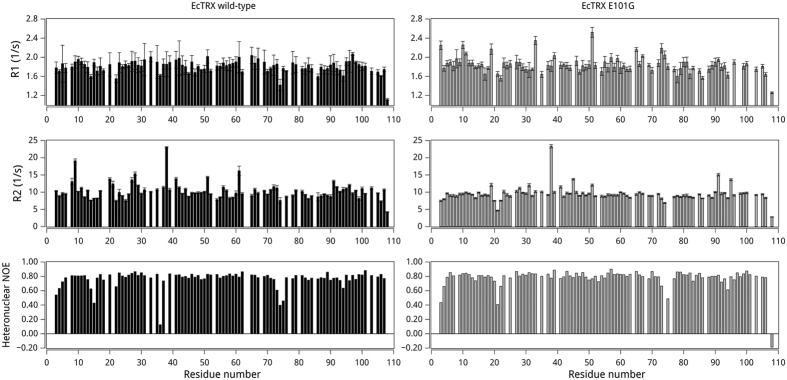
Comparison of Backbone Dynamics of Wild-Type and E101G Mutant of EcTRX by NMR. The measured ^15^N-^1^H *R*_1_ (upper panel) and *R*_2_ (middle panel) relaxation values are shown as a function of the protein sequence. The ^15^N-^1^H NOE values are shown in the lower panel.

To evaluate experimentally whether or not the E101G variant exhibits altered motions in solution, we performed standard NMR experiments to characterize the backbone dynamics of this variant. No significant alterations in internal mobility in the ps-ns timescale was observed in the heteronuclear-NOE, *T*1 and *T*2-relaxation measurements by comparison with wild-type EcTRX (Fig. 6). This suggests that altered mobility in these timescales is not the source of the conformational heterogeneity observed in the E101G crystal.

**Figure 7:**
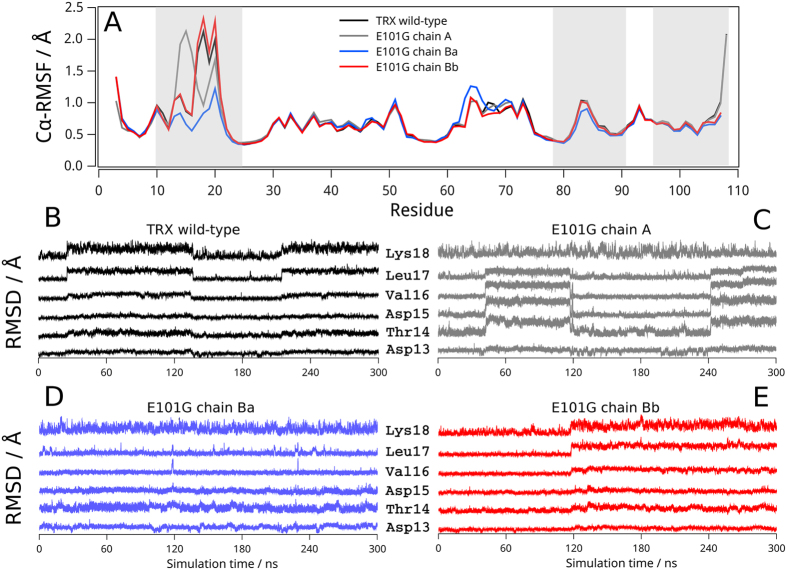
Molecular Dynamic Simulations of Wild-Type and E101G Mutant of EcTRX. The average RMSF profile over the 300 ns of simulation for wild-type (black line) and the E101G variant chain A (grey line), chain B_a_ (blue line) and chain B_b_ (red line) are shown in panel A. Plot of the time-course RMSD for residues 13–18 are presented in panels B, C, D and E for wild-type, chain A, chain B_a_ and chain B_b_, respectively. RMSF and RMSD values were calculated for backbone atoms only.

We further investigated the backbone dynamics of EcTRX in the ps-ns timescale through molecular dynamic simulations (MDs). To this end, 300 ns of classic all-atoms simulations were calculated at 298 K for chains A, B_a_ and B_b_ of the E101G mutant, and chain A of the wild type. The structural differences between all chains of the E101G mutant mostly disappeared during the energy minimization and equilibration steps. However, a remarkable difference remained between E101G conformations in the 10–25 segment (data not shown). The four conformations exhibited low mobility throughout the simulation (Fig. 7), with RMSD values of 1.2 ± 0.1 and 1.1 ± 0.1 Å for chains B_a_ and B_b_, respectively, and 1.2 ± 0.2 Å for chain A of the wild-type and the E101G variants. These low values indicate that the overall low mobility previously reported for wild-type EcTRX^13^ is not significantly perturbed upon the E101G mutation. A single segment exhibits a significantly different dynamical behavior between conformations, which is the stretch of residues 10–25. For this segment, the root-mean-square fluctuations (RMSF) for the whole simulation is lower for chain B_a_ than the other chains. For residues 13–18 in the wild-type, and E101G chains A and B_b_, a number of residues experience transitions towards significantly different conformational states, which is reflected in persistent leaps in the RMSD values (Fig. 7B–E).

One important issue is the effect of mutation on function. Previous results of our group showed that the specific activity measured by using the non-natural substrate DiFTC-insulin is 80, 95, 100, 108 and 175% for L94A, L107A, wild-type, N106A and E101G, respectively^12^. The main function of TRX is as a thiol disulfide oxidoreductase, and as such, catalysis relies on the nucleophilicity of the Cys32 thiolate and the electrophilicity of the Cys32-Cys35 disulfide.

We focused on how these two properties were affected by E101G mutation. To probe Cys32 nucleophilicity, three types of reactions were explored, namely, disulfide reduction, H_2_O_2_ reduction and alkylation. Four types of disulfides were assayed as electrophiles: one that is unspecific (hydroxyethyl disulfide, HED), two that are linked to standard TRX activity measurement (insulin and DiFTC-insulin), and one that is a natural substrate of the enzyme (EcTPx, thiol peroxidase from *E. coli*). Two alkylating agents, N-ethyl maleimide (NEM) and monobromobimane (mBBr) plus H_2_O_2_ were also studied as unspecific electrophiles. The electrophilicity of the disulfide was studied by DTT and tris-2-carboxyethyl phosphine (TCEP) reduction. Additionally, the acidity of Cys32 was measured by the change in Trp fluorescence^18^ and through the rate of reaction with mBBr^19^.

Rate constants of oxidation by insulin, HED, H_2_O_2_ and HED, alkylation by mBBr, and reduction by dithiothreitol (DTT) and TCEP were all within the 10% dispersion that is usual for this type of measurement (Table 3). Alkylation by NEM is slightly faster (27%) on E101G. Consistent with our previous report regarding an increase in enzyme activity when assayed using DiFTC-insulin^12^, the rate constant of E101G reacting with DiFTC-insulin was twice that of wild type EcTRX. Finally, the specific reduction of EcTPx was the only case in which the wild-type variant was significantly faster (50%) than E101G. Thus, the overall reactivity of either Cys32 or the Cys32-Cys35 disulfide was not greatly changed by the E101G mutation except for slight differences in unspecific reactions and somewhat larger differences where protein-protein interaction was involved, as in the case of DiFTC-insulin and, in particular, the biological substrate EcTPx.

**Figure 8:**
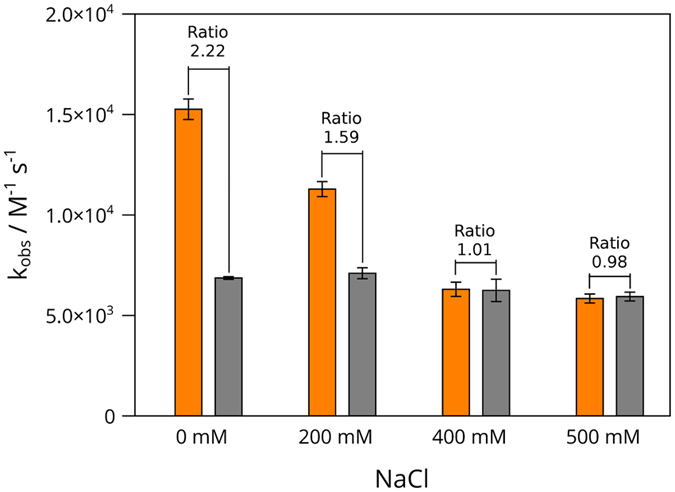
Effect of Ionic Strength on the Catalytic Rate Constant of Wild-Type and E101G Mutant of EcTRX. Activity measurements were performed as described in the Materials and Methods for DiFICT-insulin. The reaction was carried out in the presence of the indicated concentrations of NaCl. The catalytic constants obtained from each measurement are shown for wild-type (gray bar) and E101G mutant (orange bar).

Given that a negatively charged residue (Glu) is replaced by a neutral amino acid (Gly), we suspect that this difference in charge may alter the electrostatics of protein-protein interactions modifying in a subtle form the interaction with EcTPx, one of the natural targets of EcTRX, or with the non-natural substrate DiFICT-insulin, and concomitantly the EcTRX activity. It is worthy of note that each FICT moiety introduces a negative charge; therefore, two novel negative charges are present in DiFICT-insulin. To investigate whether the electrostatics of the C-terminal helix modulates the activity, we evaluated the effect of ionic strength in the catalytic rate for wild-type and E101G variants. Increasing concentrations of NaCl have no effect on the catalytic rate of wild-type EcTRX, whereas a significant decrease on the catalytic rate of E101G variant was determined (Fig. 8). Within the experimental error, both proteins showed the same activity at the highest salt concentrations assayed (400 and 500 mM), as expected for a surface charge shielding effect.

Results from Mora-Garcia and coworkers support this^20^. They showed that the activity of EcTRX E30K mutant is 30% higher than wild-type when assayed with DiFICT-insulin. This suggests that the affinity of the reduced form of EcTRX for target proteins can be modified by mutations that alter the electrostatics of the interaction surface. In fact, the increase of positive charges on the surface of EcTRX (E30K/L94K) enhances the affinity for other substrates like chloroplast fructose-1,6-bisphosphatase, and also increases specific activity. Remarkably, these effects can be shielded by ionic strength; i.e., high concentrations of KCl decrease the affinity of the highly efficient mutant EcTRX E30K/L94K^20^.

Nevertheless, given that mutant reactivity was not very different to that of the wild-type variant, it followed that the active site remained unaffected. These results were in agreement with the non-significant difference in the p*K*a values of the active site Cys residues, as measured by Trp fluorescence and Cys-alkylation with mBBr (Table 4). Similar results concerning p*K*a values were obtained for the other EcTRX mutants studied (Supplementary Table S3).

## Concluding Remarks

The three-dimensional structures of four EcTRX point mutants were determined by X-ray crystallography. The global structures were essentially unchanged, i.e., EcTRX accommodated the sequence changes and compensated the removed interactions, even tolerating voids or subtle remodeling to satisfy hydrogen bond pairing. This is in agreement with the robustness of EcTRX to sequence changes already observed for a number of mutants reported in previous works.

A number of studies have shown that the overall structure of a protein is the same in the crystal environment or in solution. In agreement with previous studies^21^,^22^, some degree of variability was observed in our structures in exposed regions, such as loops or side chains at the protein surface, due to crystal packing contacts. The consequences of crystal packing in protein structures have been examined through a detailed comparison of the structures of the same protein crystallized in several space-groups^22^,^23^. However, the differences in the crystallization conditions necessary to promote crystal growth in these alternative arrangements make it difficult to discriminate the effect of crystal packing from conformational differences originating from different solvent compositions. On the other hand, when multiple copies are found in the asymmetric unit, it is possible to explore conformational variability of a given protein by comparing individual monomers, irrespective of solvent composition or differences in crystal-space groups. It was argued that modest albeit significant conformational differences observed in crystal structures of a given protein (obtained in similar conditions) represent alternative substates of the native-state ensemble with concomitantly small energy barriers, comparable in magnitude to the ones that separate these near-iso-energetic substates in solution^22^. Thus, keeping in mind that point mutation E101G minimally perturbs the free-energy profile of wild-type EcTRX, and even when the abundance in solution of each one of the conformations detected by X-ray for EcTRX cannot be established, evidence for unambiguous alternative conformations allows a more suitable representation of the EcTRX structure, provided by the ensemble. Given the fact that the occurrence of alternative traces in the E101G crystal was not concurrent with particularly high or low packing density, a delicate balance of packing forces jointly with slight perturbations of the energetics of the native state may be responsible for stabilization of these alternative conformations.

The alternative conformation observed for the backbone of the E101G variant conform, to our knowledge, the widest region with alternative traces for the backbone reported to date. It is direct evidence by X-ray crystallography for conformational variability in the EcTRX family, regardless of the spatial group or differences in crystallization conditions. With the exception of the stretch 10–25, the variable regions in this crystal are not correlated with either the most flexible regions in MDs or the most structurally diverse segments in a set of deposited structures of EcTRX. Additionally, as no significant changes in dynamic behavior were observed upon the E101G mutation, and enzyme activity remained essentially identical for most of the assayed substrates, it followed that the presence of alternative traces was not the consequence of large conformational changes of the mutant in solution. The set of mutants investigated in this paper represented an opportunity to explore by X-ray some structural properties shared with the wild-type EcTRX.

## Material and Methods

### Purification and Crystallization of TRX Variants

The EcTRX variants L94A, E101G, N106A and L107A were produced in *Escherichia coli* and purified as previously reported^12^. Prior to crystallization, EcTRX were extensively dialyzed against deionized water and concentrated. Crystallization was carried out using the hanging drop method at 16 °C. Well-diffracting crystals were obtained as follows. For variant L94A, the drop was a 1:1 mix of protein (10 mg/ml) and reservoir solution (100 mM sodium acetate, 4 mM CuSO_4_, 18% ethanol, pH 4.6). For variant E101G, the drop was a 1:1 mix of protein (18 mg/ml) and reservoir solution (100 mM sodium acetate, 4 mM CuSO_4_, 15% ethanol, pH 4.75). For variant N106A, the drop was a 1:1 mix of protein (10 mg/ml) and reservoir solution (100 mM sodium acetate, 4 mM CuSO_4_, 25% ethanol, pH 4.25). For variant L107A, the drop was a 2:1 mix of protein (10 mg/ml) and reservoir solution (100 mM sodium acetate, 4 mM CuSO_4_, 18% ethanol, pH 4.25).

### X-ray Data Collection and Processing, Structure Determination and Refinement

Prior to data collection, crystals were cryoprotected in the same buffers as reservoir solutions plus 30% glycerol and then flash-cooled in liquid nitrogen. Diffraction data were collected at 100 K on the X06DA beamline at the Swiss Light Source (SLS) (Switzerland) equipped with a Pilatus 2 M detector (Dectris Ltd., Baden, Switzerland). Indexing, integration and scaling were performed using the HKL2000 suite of programs^24^. Five percent of the measured reflections were flagged for cross-validation^25^. Relevant statistics of data collection and processing are given in Table 1.

The X-ray structures were determined by molecular replacement (MR) using the coordinates of wild-type EcTRX (PDB ID: 2TRX) for variants E101G and L107A or the double mutant M37L/P40S (PDB ID: 1KEB) for variants L94A and N106A. For both the E101G and L107A variants, MR was performed using Phaser^26^ as implemented in the MrBump pipeline^27^ from the CCP4 suite^28^, whereas in the case of the L94A and N106A variants, MR was carried out using Phaser but implemented in the Phenix suite^29^. Refinement was performed using REFMAC5^30^ and Coot^31^. Additionally, a round of refinement using the server PDBREDO^32^ resulted in a significant improvement in fitting the model to data for the L94A and L107A structures. Final refinement was carried out using Phenix for all the structures. Hydrogen atoms were modelled at riding positions, and anisotropic B-factors of protein atoms were employed for the L94A, E101G and N106A structures. For the L107A structure, B-factors were treated using TLS refinement, with one TLS group per protein chain. The stereochemical qualities of models were verified using the Molprobity server^33^ and the validation server of the RCSB PDB. The coordinates and structure factors were deposited in the PDB (PDB entries 5HR0, 5HR1, 5HR2 and 5HR3 for E101G, L107A, L94A, and N106A variants, respectively).

### Determination of the Acidity Constant of the TRX Wild Type and E101G Variants

The proteins were reduced during 1 hour using 10.0 mM DTT and then the DTT was removed by gel filtration. Each protein (3.8 μM) was placed in a 96-well plate at 12 different pH-values in a buffer consisting of 30 mM ACES, 15.6 mM ethanolamine, 15.6 mM, TRIS, 120 mM NaCl and 0.1 mM DTPA, adjusted using HCl or NaOH to obtain the desired pH value (from pH 2.6 to 10.1). Each condition was measured by quadruplicate. The reaction was started by the addition of 1 μM (at pH > 7) or 2 μM (at pH < 7) monobromobimane (mBBr), and time-traces of fluorescence were recorded simultaneously at all pH-values for at least 30 minutes in a Varioskan Flash microplate reader (Thermo Scientific). The excitation wavelength was set at 396 nm and emission was 482 nm. A two-pK_a_ model was fitted to the experimental data, which contemplated three possible reactions, one with each protonation state of the proteins, as previously described^19^.

Alternatively, the pK_a_s were measured by the fluorimetric titration of the protein. Briefly, each TRX variant (3 μM) was placed in a buffer containing acetic acid (15 mM), MES (15 mM), Tris (30 mM), dtpa (100 μM) and DTT (2 mM) with pH adjusted to 8.8 using NaOH. The resulting solution was titrated by the addition of small volumes (2–4 μL) of 2 M HCl with stirring. After each addition, pH was measured and the emission spectrum (300–400 nm, λ_ex_ = 280 nm) was recorded. The maximal emission vs. pH plot was fitted to a two-pK_a_ equation; additionally, the center of mass of the emission in the 300 to 400 nm range was also plotted vs. pH and fitted to a single pK_a_ equation.

### Measurement of Kinetic Rate Constants for Wild-type EcTRX and Variant E101G

Nucleophilicity of the wild-type and E101G variants was measured as the rate constant of their reaction with monobromobimane (mBBr), N-ethyl maleimide (NEM), hydrogen peroxide (H_2_O_2_), hydroxyethyl disulfide (HED), bovine insulin (Ins), Di-fluoresceinthiocarbamyl-insulin (DiFTC-Insulin) and oxidized TPX from *E. coli* (EcTPx).

EcTRX (wild-type or E101G) was reduced during 1 hour using 10.0 mM DTT and then the DTT was removed by gel filtration using a HiTrap desalting column (GE Healthcare) in an ÄKTA prime plus chromatography system (GE Healthcare). The content of reduced cysteine was determined spectrophotometrically by disulfide reduction using 4,4′-dithiodipyridine (ε_324 nm_ = 21400 M^−1 ^cm^−1^)^34^.

Unless otherwise indicated, the reactions were performed in a stopped flow device coupled to a fluorometer (Cary Eclipse, Agilent Technologies) using a 1:1 mixing ratio (final volume of 300 μL), 1.5 μM of EcTRX and at least a tenfold excess of the other reagent under pseudo-first order conditions. The time course of TRX fluorescence was recorded with excitation wavelength set at 280 nm and emission at 345 nm in most cases. For alkylation with mBBr, the fluorescence of the product (λ_ex_ 396 nm, λ_em_ 482 nm) was used for monitoring the reaction. In the case of a reduction of DiFTC-insulin, the reaction was performed under pseudo-first order conditions with 100 nM DiFTC-insulin and TRX as the excess reagent; the time course of fluorescein fluorescence (λ_ex_ 495 nm, λ_em_ 519 nm) was recorded^35^,^36^.

Reduction of TRX by dithiothreitol (DTT), tris-2-carboxyethyl phosphine (TCEP), was performed under pseudo-first order conditions with the reductant in excess. TRX disulfide (2 μM) reacted with at least a tenfold excess of DTT or TCEP, and the time course of TRX fluorescence was recorded.

All time courses were fitted to a single exponential function and the pseudo-first order rate constant thus obtained was plotted vs. the concentration of the reagent in excess to obtain the second order rate constant.

In all cases, the reaction buffer was 15 mM acetic acid, 30 mM TRIS, 15 mM MES, 120 mM NaCl and 0.1 mM dtpa at pH 7.2. Preparation and quantification of the substrates were performed as previously described.

### NMR Spectroscopy

^15^N-labeled proteins were obtained from *E. coli* cultures grown in M9 minimal medium, supplemented with ^15^N-NH_4_Cl (Cambridge Isotope Laboratories, Andover, USA) and purified as the non-labelled samples. Spectra were acquired at 20 °C in a Bruker 600 MHz Avance II spectrometer (Bruker Instruments Inc., Billerica, USA). Protein concentration was 400 μM and the buffer was 20 mM Tris-HCl, 100 mM NaCl, 1 mM DTT, pH 7.0, supplemented with 10% D_2_O. The relaxation data *R*1, *R*2, and ^1^H-^15^N steady-state NOEs were measured using standard sequence pulses^37^.

### Structural Analysis

Intramolecular interactions were computed with the PIC server^38^. The Bio3d^39^ package was used for structure superposition and RMSD calculations. Crystal packing was calculated after generation of symmetry-related molecules in pymol, counting the number of intermolecular contacts using the *list_contacts.py* command from Robert L. Campbell’s script repository:

http://pldserver1.biochem.queensu.ca/~rlc/work/pymol/

For RMSD calculations in a set of EcTRX structures, annotated EcTRX structures were retrieved from the PDB, pruned to chain A, and superposed. Very divergent or highly substituted structures were removed, and the A chains of the four variants determined in this work were added to the set. All possible pairwise structural alignments and corresponding RMSD calculations were generated in Bio3d, and the average and standard deviation of these values were calculated. The PDB IDs of the dataset of previously reported EcTRX structures are: 1KEB, 1TXX, 1ZZY, 2EIO, 2EIR, 2FCH, 2FD3, 2H6X, 2H6Y, 2H6Z, 2H70, 2H71, 2H72, 2H73, 2H74, 2H75, 2H76, 2TIR, 2TRX and 3DYR.

### Molecular Dynamic Simulations

The atomic coordinates of wild type TRX from *E. coli* (PDB: 2TRX, chain A) and the A, B_a_ and B_b_ conformations of the E101G variant (PDB: 5HR0) were solvated with the 3-particle TIP3P model^40^ in an orthorhombic box, which extended 15 Å farther from the nearest protein atom using the *ff14SB* force field^41^. To neutralize the systems, sodium counter ions were added to balance the charge of the protein. Minimization was applied to the resulting structure to remove any clashes for 1,000 steps of steepest descent followed by 1,000 steps of conjugate gradient minimization using constant-volume periodic boundary conditions. The system was heated up from 10 K to 298 K, using the Berendsen thermostat^42^ with a time constant of 2 ps and subsequently switched to constant isotropic pressure to allow density to equilibrate.

Production simulations were performed using the Monte Carlo barostat and the Langevin thermostat at a collision frequency of 5 ps^−1^. The SHAKE algorithm^43^ was applied to all bonds involving hydrogen atoms, then 2 fs time step was settled. An 8 Å cut-off radius for range-limited interactions with particle mesh *Ewald* summation for long-range electrostatic interactions was used. Harmonic positional restraints at a strength of 20 kcal mol^−1 ^Å^−2^ on Cα-atoms were applied during minimization and equilibration. Restraints were removed in 5 successive simulation stages (20, 10, 5, 2 and 0 kcal mol^−1 ^Å^−2^) of 5 ns each. Unrestrained molecular dynamic simulations were performed with the *pmemd*.CUDA engine in the Amber14^44^ suite for the GPU code. Data analysis was performed using the *cpptraj* module of AmberTools15^45^.

## Additional Information

**How to cite this article:** Noguera, M. E. *et al*. Structural variability of *E. coli* thioredoxin captured in the crystal structures of single-point mutants. *Sci. Rep.* **7**, 42343; doi: 10.1038/srep42343 (2017).

**Publisher's note:** Springer Nature remains neutral with regard to jurisdictional claims in published maps and institutional affiliations.

## Supplementary Material

Supplementary Information

## Figures and Tables

**Figure 1:**
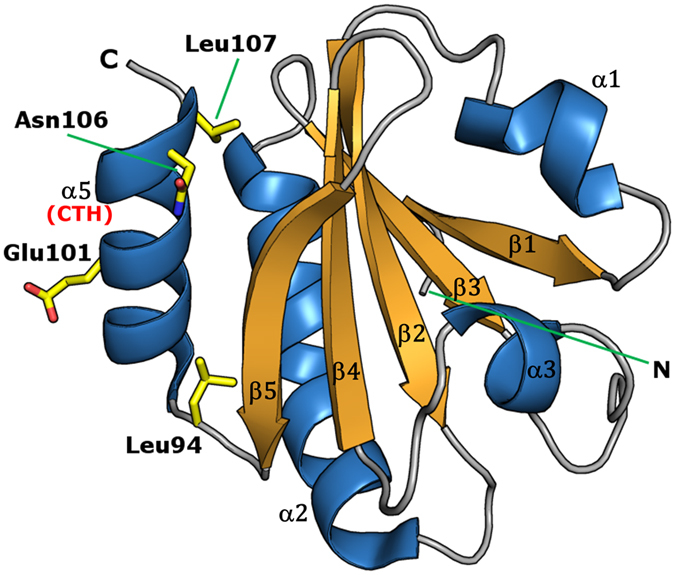
The Three-Dimensional Structure of EcTRX. The X-ray structure of wild-type EcTRX (PDB: 2TRX) is shown as a ribbon representation. In addition, the side chains of the mutated residues studied in this work are shown as sticks.

**Table 1:** Data Collection and Refinement Statistics.

	L94A	E101G	N106A	L107A
DATA COLLECTION				
Wavelength (Å)	0.9202	0.9191	0.9202	0.9202
Space Group	C2	P3_1_21	P1	P2_1_2_1_2_1_
Unit Cell Parameters				
*a* (Å)	87.0	50.0	29.2	85.4
*b* (Å)	47.9	50.0	33.1	88.7
*c* (Å)	28.9	163.3	47.2	115.5
*α* (°)	90.0	90.0	75.8	90.0
*β* (°)	102.0	90.0	88.9	90.0
*γ* (°)	90.0	120.0	68.8	90.0
Resolution Limits (Å)	28.25–1.20	19.86–1.31	16.23–1.10	38.48–2.15
	(1.26–1.20)	(1.36–1.31)	(1.14–1.10)	(2.23–2.15)
*R*_*merge*_	0.057 (0.087)	0.055 (0.451)	0.036 (0.242)	0.065 (0.515)
Mean I/σ (I)	11.9 (6.3)	22.1 (4.1)	20.2 (3.2)	25.3 (4.2)
Completeness (%)	94.3 (81.3)	99.6 (99.9)	93.00 (89.20)	99.2 (99.3)
Multiplicity	3.3 (3.0)	5.4 (5.0)	2.00 (1.90)	6.5 (6.7)
*V*_M_ (Å^3^ Da^−1^)	2.50	2.51	1.75	2.66
Solvent Content (%)	50.9	51.1	29.78	53.71
No. Molecules in ASU	1	2	2	7
REFINEMENT				
Resolution Limits (Å)	28.25–1.20	19.86–1.31	16.23–1.10	38.48–2.15
Number of Reflections	34197	57845	59917	48297
*R*_*work*_/*R*_*free*_	0.1318/0.1515	0.1739/0.2092	0.1419/0.1634	0.2064/0.2348
No. Protein Atoms	821	1964	1644	5439
No. Water Molecules	125	170	187	100
Deviations from Ideal Geometries				
Bond Length (Å)	0.011	0.011	0.011	0.007
Bond Angles (°)	1.122	1.095	1.203	0.574
Ramachandran Plot				
Most Favored (%)	99.1	99.6	99.1	99.9
Allowed (%)	0.9	0.4	0.9	0.1

Values in parentheses refer to the highest resolution shell.

**Table 2:** Intramolecular Interactions within each Conformation of the EcTRX E101G Variant.

	Chain A	Chain B_a_	Chain B_b_
Hydrophobic Interactions within 5 Å	114	113	114
Main Chain-Main Chain Hydrogen Bonds	126	124	124
Main Chain-Side Chain Hydrogen Bonds	23	20	24
Side Chain-Side Chain Hydrogen Bonds	11	14	9
Ionic Interactions within 6 Å	8	9	9
Aromatic-Aromatic Interactions within 4.5 and 7 Å	5	5	5
Aromatic-Sulphur Interactions within 5.3 Å	0	1	1
Cation-Pi Interactions within 6 Å	1	2	3

Interactions were calculated using the PIC server^38^.

**Table 3:** Reactivity of TRX Wild-Type and E101G in Oxidation, Alkylation and Reduction Reactions.

TRX	Reaction		Second order rate constant, 25 °C, pH 7 (M^−1 ^s^−1^)	
			WT	E101G
Reduced	Oxidation	Insulin^(a)^	2.30 ± 0.02 × 10^4^	2.56 ± 0.03 × 10^4^
		DiFTC-Insulin^(b)^	7.8 ± 0.6 × 10^3^	13.9 ± 0.7 × 10^3^
		EcTPx^(a)^	8.9 ± 0.4 × 10^4^	5.9 ± 0.3 × 10^4^
		HED^(a)^	9.90 ± 0.13	11.3 ± 0.4
		H_2_O_2_^(a)^	2.20 ± 0.04	2.30 ± 0.16
	Alkylation	mBBr^(c)^	37.0 ± 1.2	39.0 ± 1.9
		NEM^(a)^	3.04 ± 0.03 × 10^3^	3.88 ± 0.03 × 10^3^
Oxidized	Reduction	DTT^(a)^	247 ± 2	254 ± 2
		TCEP^(a)^	3.0 ± 0.3	3.0 ± 0.2

^(a)^Monitored by Trp fluorescence λ_ex_ = 280 nm λ_em_ = 345 nm.

^(b)^Monitored by fluorescein fluorescence λ_ex_ = 495 nm and λ_em_ = 519 nm.

^(c)^Monitored by bimane thioether fluorescence λ_ex_ = 396 nm and λ_em_ = 482 nm.

**Table 4:** Cys32 p*K*_a_ Measured for Wild-Type and E101G TRX.

Method of Determination	TRX Variant	
	Wild-Type	TRX E101G
Rate of alkylation by mBBr *vs* pH^(a)^	7.3 ± 0.3 (9.3 ± 0.3)^(b)^	6.80 ± 0.20 (9.0 ± 0.2)^(b)^
Trp fluorescence (Intensity *vs* pH)^(a)^	(6.00 ± 0.04)^(c)^ 7.5 ± 0.2	(5.91 ± 0.07)^(c)^ 7.1 ± 0.1
Trp fluorescence (emission center of mass *vs* pH)	7.23 ± 0.06	7.10 ± 0.04

^(a)^A two-p*K*_a_ function was fitted to the data.

^(b)^Assigned to Cys35.

^(c)^Assigned to an unknown residue ionization that affects Trp emission intensity.
