# Supplementary material for: Structural variability of E. coli thioredoxin captured in the crystal structures of single-point mutants
Source: Sci Rep. 2017 Feb 9;7:42343. doi: 10.1038/srep42343 (PMC5299410; doi:10.1038/srep42343)
Supplement: Supplementary Information [file srep42343-s1.pdf]

Structural variability of *E. coli* thioredoxin captured in the crystal structures of single point mutants

Supplementary Material

Martín E. Noguera,[1,§] Diego S. Vazquez,[1,§] Gerardo Ferrer-Sueta,[2] William A. Agudelo,[1] Eduardo Howard,[3] Rodolfo Rasia,[4] Bruno Manta[2], Alexandra Cousido-Siah,[3] André Mitschler,[3] Alberto Podjarny,[3] and Javier Santos[1,]*

[1]Universidad de Buenos Aires, Facultad de Farmacia y Bioquímica, Instituto de Química y Fisicoquímica Biológicas, CONICET, Junín 956, C1113AAD, Buenos Aires, Argentina

[2] Laboratorio de Fisicoquímica Biológica, Instituto de Química Biológica and Center for Free Radical and Biomedical Research, Universidad de la República, Montevideo, Uruguay.

[3]Department of Integrative Biology, *IGBMC, CNRS, INSERM, Université de Strasbourg, Illkirch, France.*

[4]Instituto de Biología Molecular y Celular de Rosario (IBR), Rosario, Santa Fe, Argentina.

[§] These authors contributed equally to this work.

[*] Correspondence should be addressed to: javiersantosw@gmail.com.

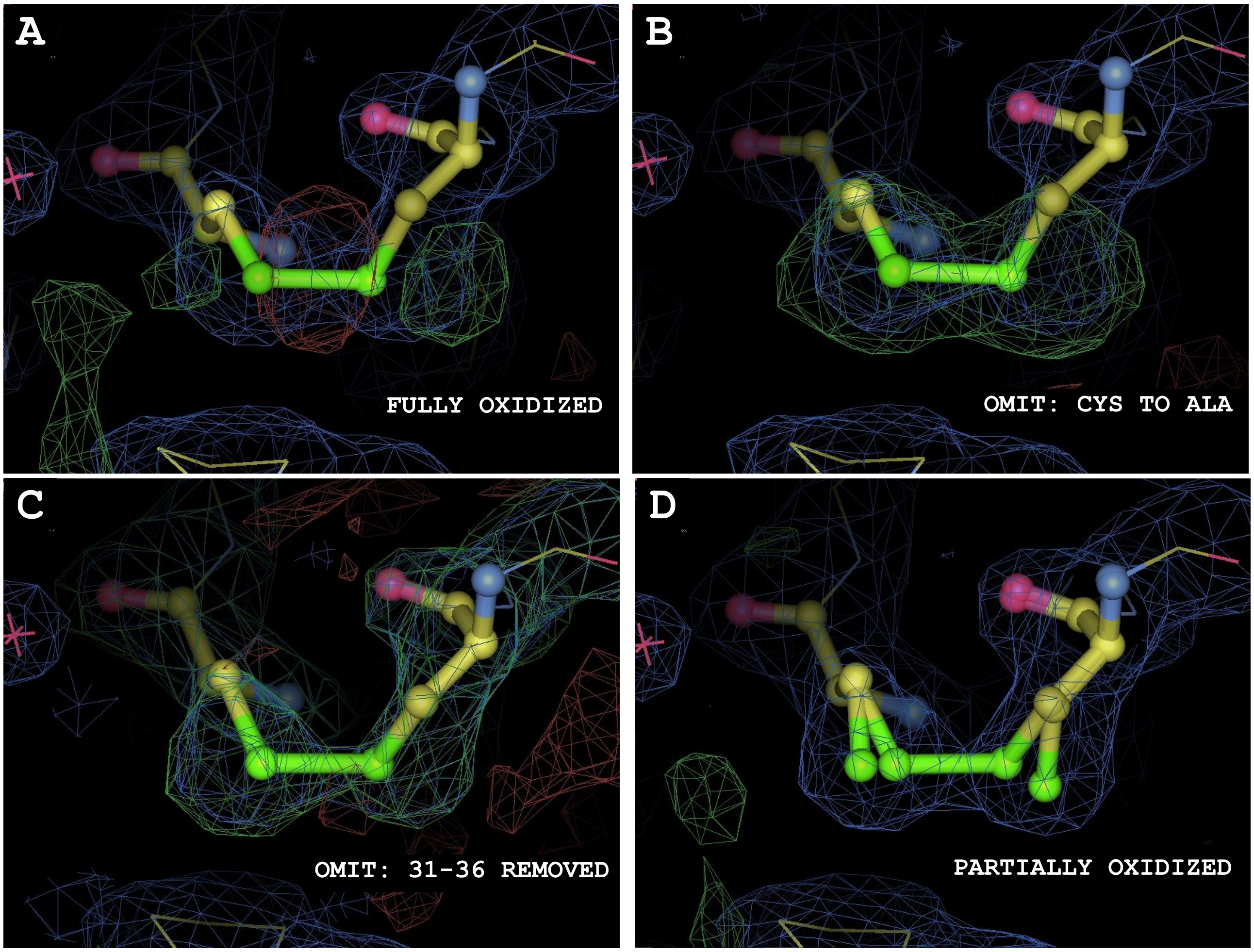

Figure S1. Redox-state of cysteine residues. (A) A close examination of the final F_o-F_c electron density map revealed significant negative density between the Sγ atoms of some disulfide bonds (red) and additional positive density in the opposite sides of Sγ atoms (green). Close-up of the Cys32-Cys35 disulfide pair of chain A in the L107A variant, one of the most affected cases. $2F_o$-F_c electron density maps are contoured at the 1.5σ level (blue), and F_o-F_c contoured at 3.0σ level. Cysteine residues are depicted as balls and sticks. (**B**) An omit map was calculated mutating each cysteine to alanine in the final (oxidized) model of the L107A variant, followed by refinement in Phenix as described in the Materials and Methods section, plus the Torsion-based Simulated Annealing option to reduce phase bias. The molecular model corresponding to the fully-oxidized refinement is shown overlaid to the omit map. (**C**) Residues 31-36 were removed in the final (oxidized) model and a new round of refinement was calculated in the same way as described in (B). The molecular model of the fully-oxidized refinement is shown overlaid to the omit map. (**D**) Alternative conformations for cysteines were added to the final (oxidized) model, and a new round of refinement was carried out as described for panel (B), but cysteines in alternative conformations were constrained to the reduced state.

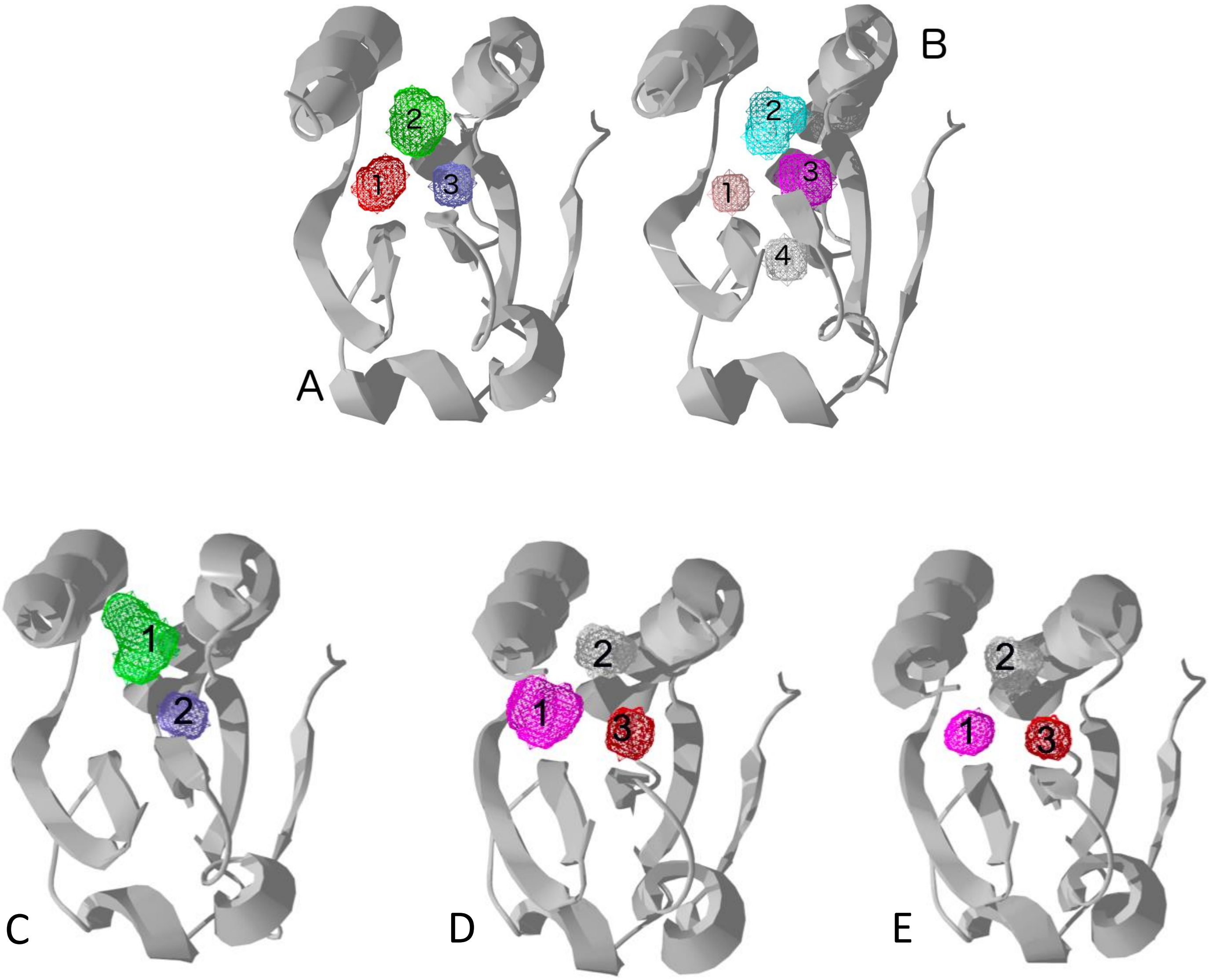

Figure S2. Cavities in EcTRX. Wild-type EcTRX Chain A (A) and chain B (B) and E101G variant chain A (C), chain Ba (D) and chain Bb (E) are shown with the corresponding cavities found using Swiss PDB viewer with default parameters.

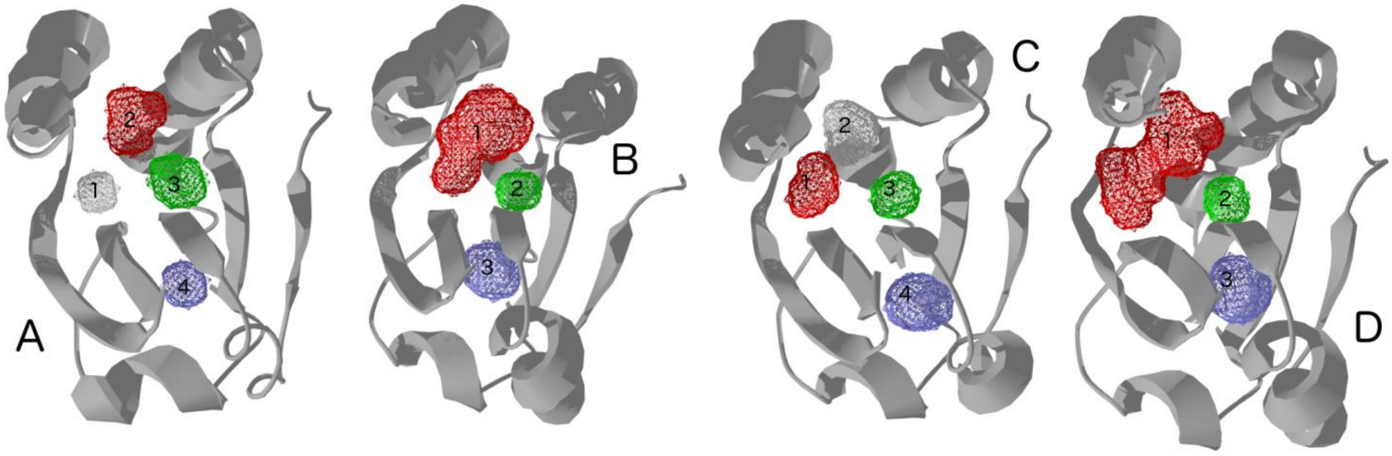

Figure S3. **Comparison of cavities found in Ec-TRX variants.** (A) Chain B from wild-type Ec-TRX; (B) variant L94A; (C) chain A from variant L107A; (D) chain B from variant N106A structure. Cavities are described using the same color and numbering nomenclature as in **Table S1**. Regarding the volume and area of each cavity, some heterogeneity for the different chains in L107A structure (data not shown) was evidenced.

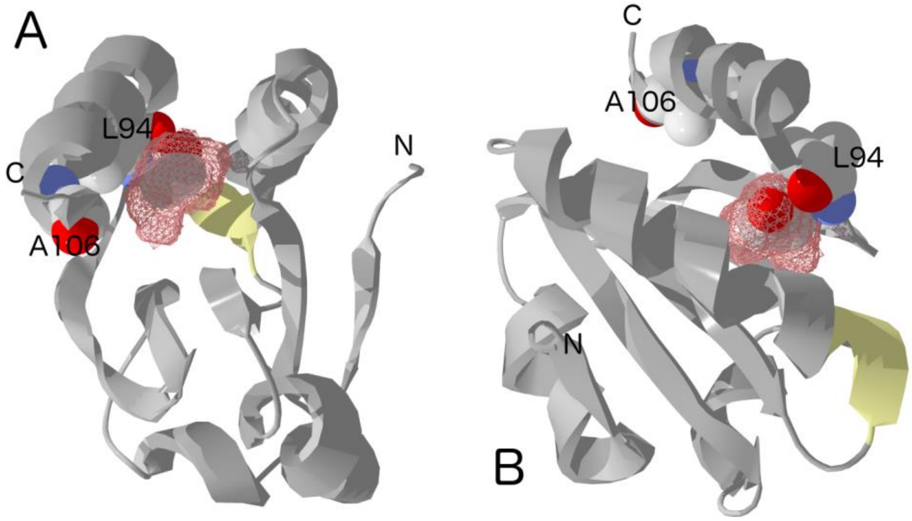

Figure S4. An ethanol molecule is present in the large cavity of N106A variant. (A) and (B) represent two different views (turning ~120º). The large cavity found using a Swiss PDB viewer with default parameters in chain B is represented using plain lines in red. The putative ethanol molecule, assigned by the electron density observed inside the cavity is shown using CPK representation

(excluding the hydrogen atoms for clarity). The oxygen from the -OH group is at a distance of 2.8 Å from the carbonyl oxygen of L94. The site of mutation (residue A106) is shown. In addition, the active site of TRX (-C_{32}-G-P-C_{35}-) is shown in yellow (ribbon representation)

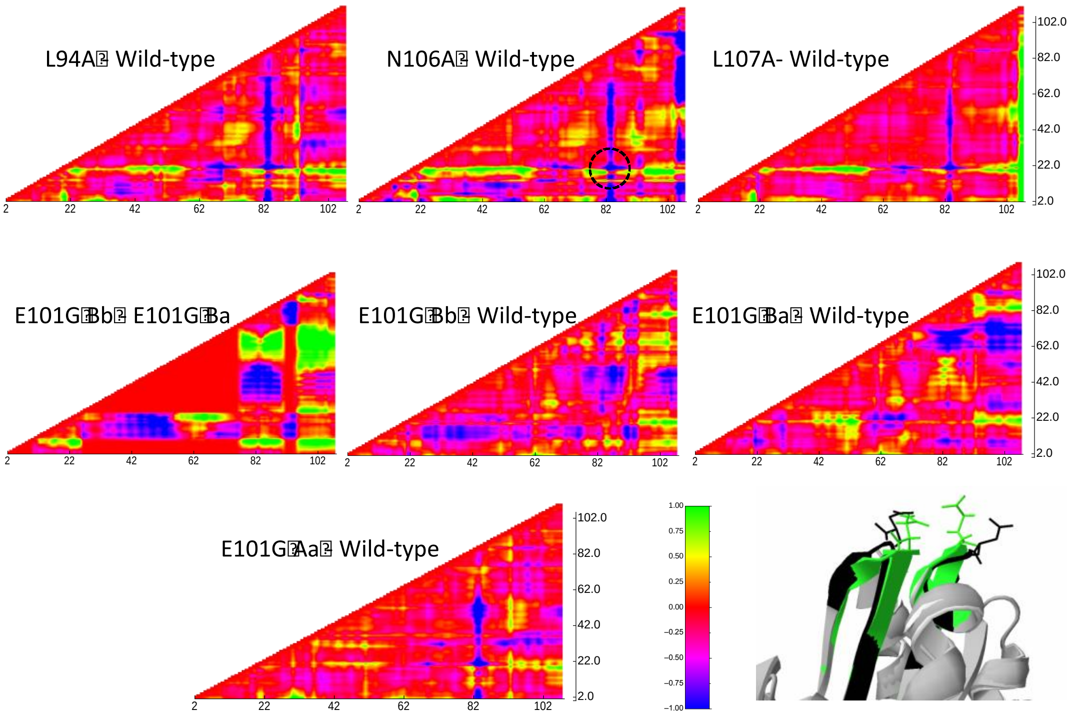

Figure S5. Structure comparisons by difference distance plots using the alpha carbon atoms. For each structure a Cα-Cα distance matrix was computed. After that, the per-residue difference in distance values between pairs of structures was plotted as indicated. The analysis was performed using the Bio3d package. The same color scale was used in all cases and residue numbering correspond to PDB ID: 2TRX. An example of the local differences observed is shown for N106A and wild-type. As explained in the main text, segments centered in residues 20 and 83 exhibit differences in internal distances (a dashed circle in the upper central panel). A ribbon representation is shown on the right, bottom (wild-type and N106A variants are shown in black and green, respectively and residues N83 and D20 are shown in sticks).

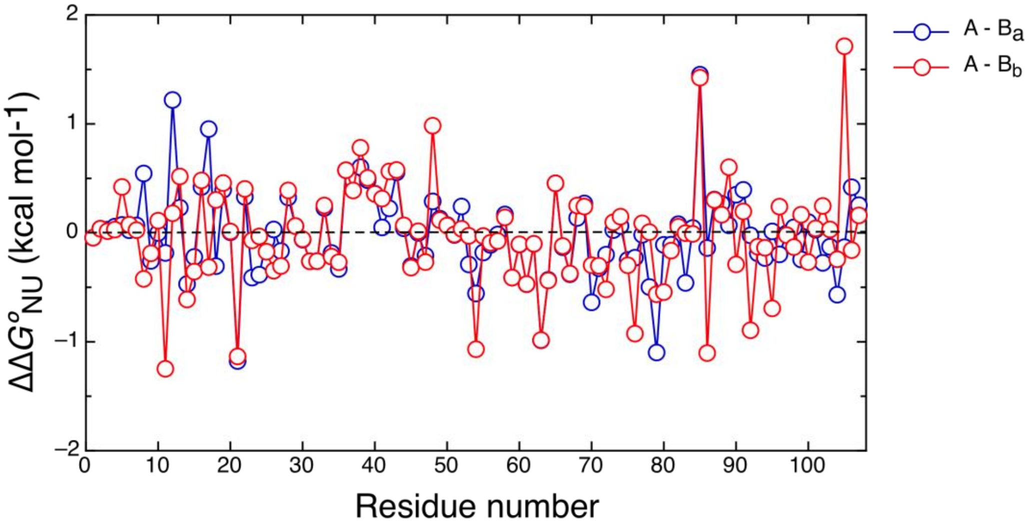

Figure S6. *FoldX* calculations were performed for conformations of chain A and chains B_a and B_b of the E101G mutant after applying the repair protocol. The $\Delta\Delta G^o_{NU}$ per residue between conformations A and B_a ($\Delta G^o_{NU\ A}$ - $\Delta G^o_{NU\ Ba}$) and conformations A and B_b ($\Delta G^o_{NU\ A}$ - $\Delta G^o_{NU\ Bb}$) are shown. Free energy values per residue for each conformation were obtained carrying out the sequence detail protocol.

Table S1. Relevant cavities found in EcTRX variants.

Variant	Cavity	Å^2	Å^3
**EcTRX (chain A)**			
	1 (red)	50	28
	2 (green)	70	44
	3 (blue)	34	16
**EcTRX (chain B)**			
	1 (salmon)	33	16
	2 (cyan)	70	40
	3 (fuchsia)	52	30
	4 (grey)	33	16
**E101G (chain A)**			
	1 (green)	91	61
	2 (blue)	39	19
**E101G (chain Ba)**			
	1 (fuchsia)	83	56
	2 (grey)	51	30
	3 (red)	42	221
**E101G (chain Bb)**			
	1 (fuchsia)	39	20
	2 (grey)	54	31
	3 (red)	38	19
**L107A (chain A)**			
	1 (red)	60	37
	2 (grey)	60	37
	2 (green)	42	22
	3 (blue)	59	36
**L94A**			
	1 (red)	183	135
	2 (green)	38	19
	3 (blue)	61	38

Variant	Cavity	Å^2	Å^3
**N106A (chain A)**			
Occupied cavity	1 (red)	166	137
	2 (green)	38	19
	3 (blue)	56	34
**N106A (chain B)**			
Occupied cavity	1 (salmon)	130	101
	2 (cyan)	33	16

Table S2. FoldX calculations for conformations A, B_a and B_b. Prior to FoldX calculation, a standard Repair protocol was performed for each conformation.

**FoldX Summary**	**A**	**B_a**	**B_b**
Back Bone H-Bond	-67.96	-67.86	-63.56
Side Chain H-Bond	-21.6	-18.66	-17
Energy VDW	-114.75	-115.92	-115.65
Electro	-1.72	-1.35	-1.02
Energy Solvp	145.92	144.88	144.12
Energy Solvh	-161.31	-163.86	-164.33
Energy VDW Clash	1.08	4.17	1.78
Energy Torsion	2.24	2.85	1.95
Backbone VDW Clash	55.55	58.31	61.2
Entropy Side Chain	57.64	56.49	55.78
Entropy Main Chain	149.04	148.07	147.71
Water Bonds	0	0	0
Helix Dipole	-0.34	0.43	1
Loop_Entropy	0	0	0
Cis Bond	1.12	1.12	1.12
Disulfide	-1.35	-0.88	-0.88
Kn Electrostatic	0	0	0
Partial Covalent Interactions	0	0	0
Energy Ionisation	0.05	0.05	0.05
Entropy Complex	0	0	0
Total	***-11.94***	***-10.46***	***-8.93***

Table S3. Cys32 and Cys35 pK_a Measured for Wild-Type and Mutants. p*K*a values were determined by using the rate of alkylation by mBBr *vs* pH method. A two-pK_a function was fitted to the data.

**EcTRX Variant**		
Variant	Cys32	Cys35
WT	7.3 ± 0.3	9.0 ± 0.3
L94A	6.9 ± 0.1	8.8 ± 0.1
E101G	6.8 ± 0.2	9.0 ± 0.2
N106A	6.5 ± 0.1	8.9 ± 0.1
L107A	7.3 ± 0.1	ND*

* not determined
